# Differential modulation of thalamo-parietal interactions by varying depths of isoflurane anesthesia

**DOI:** 10.1371/journal.pone.0175191

**Published:** 2017-04-06

**Authors:** Dongrae Cho, Teo Jeon Shin, Jinsil Ham, Dong-Hyuk Choi, Seonghyun Kim, Seongwook Jeong, Hyoung-Ihl Kim, Jae Gwan Kim, Boreom Lee

**Affiliations:** 1 Department of Biomedical Science and Engineering, Institute of Integrated Technology, Gwangju Institute of Science and Technology, Gwangju, South Korea; 2 Department of Pediatric Dentistry and Dental Research Institute, School of Dentistry, Seoul National University, Seoul, Korea; 3 Department of Anesthesiology and Pain Medicine, Chonnam National University Medical School, Gwangju, South Korea; Centre National de la Recherche Scientifique, FRANCE

## Abstract

The thalamus is thought to relay peripheral sensory information to the somatosensory cortex in the parietal lobe. Long-range thalamo-parietal interactions play an important role in inducing the effect of anesthetic. However, whether these interaction changes vary with different levels of anesthesia is not known. In the present study, we investigated the influence of different levels of isoflurane-induced anesthesia on the functional connectivity between the thalamus and the parietal region. Microelectrodes were implanted in rats to record local field potentials (LFPs). The rats underwent different levels of isoflurane anesthesia [deep anesthesia: isoflurane (ISO) 2.5 vol%, light anesthesia (ISO 1 vol%), awake, and recovery state] and LFPs were recorded from four different brain areas (left parietal, right parietal, left thalamus, and right thalamus). Partial directed coherence (PDC) was calculated for these areas. With increasing depth of anesthesia, the PDC in the thalamus-to-parietal direction was significantly increased mainly in the high frequency ranges; however, in the parietal-to-thalamus direction, the increase was mainly in the low frequency band. For both directions, the PDC changes were prominent in the alpha frequency band. Functional interactions between the thalamus and parietal area are augmented proportionally to the anesthesia level. This relationship may pave the way for better understanding of the neural processing of sensory inputs from the periphery under different levels of anesthesia.

## Introduction

General Anesthesia is defined as the state in which reversible loss of consciousness, sensation, motor, and reflex function are induced. In order to be able to perform surgery associated with highly noxious stimuli, general anesthetics should have hypnotic, amnesic, and analgesic effect. Although the mechanism of unconsciousness during anesthesia has been extensively investigated in previous studies [1–3], the neurophysiological mechanisms of the analgesic effect induced by anesthesia have attracted little interest.

Recently, the functional role of the thalamus as a consciousness switch has gained attention as a putative process underlying the hypnotic and amnesic effect of anesthetics [4]. However, a large body of evidence has shown that the thalamus processes sensory information from the periphery and relays it to the sensory cortex [5]. The integration of information between two brain areas is necessary to information processing [6,7]. Therefore, we infer that many anesthetics can modulate the interaction between the thalamus and the sensory cortex. In agreement with this hypothesis, several lines of evidence suggest that anesthetics alter thalamo-cortical functional connectivity [3,8,9]. High concentrations of anesthetics are usually administered before highly invasive surgical procedures because it is believed that a deep level of anesthesia is needed to deal with the elevated surgical stress. This procedure suggests that the level of anesthetic depth may affect sensory processing, therefore affecting the responses to noxious stimuli. However, to our knowledge, the effects of varying depths of anesthesia on the interaction between the thalamus and the parietal area have not been fully investigated.

Therefore, in the present study, we investigate how different levels of anesthesia induced by a volatile anesthetic (e.g. isoflurane) affect the interaction between the thalamus and the parietal region.

## Materials and methods

### Microelectrode implantation

**Fig 1:**
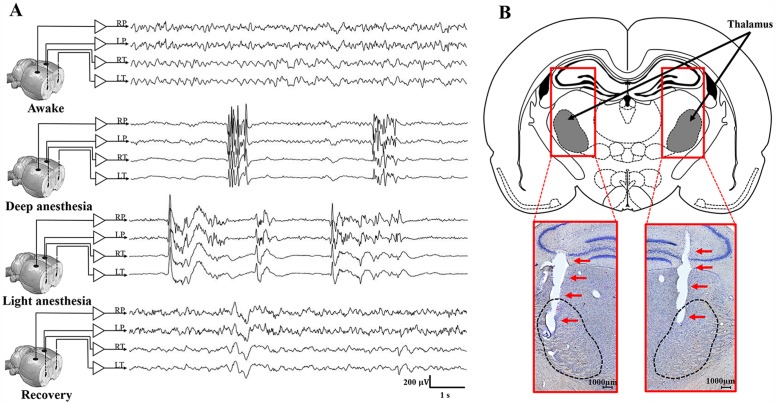
LFP recording under the four different stages and the locations of deep microelectrode in the thalamus. (A): LFPs were recorded from the right parietal (RP), left parietal (LP), right thalamus (RT) and left thalamus (LT) in the awake, deep anesthesia, light anesthesia and recovery stages. (B): Deep electrodes (red arrows) reached bilateral thalami (black dash line).

All animal experiments complied with the institutional guidelines of the Gwangju Institute of Science and Technology (GIST). All procedures were approved by the Institutional Animal Care and Use Committee of GIST. Ten male Long-Evans rats that weighted 504 ± 106 g (mean ± standard deviation) were used for this experiment. The rats were single-caged in a controlled animal facility at 21 ± 1°C with water *ad libitum*. All animal care units were preserved on a 12-hour light-dark cycle with light on at 7:00 h. The microelectrodes were implanted in the parietal lobe and thalamic region in order to measure local field potentials (LFP). The rat was anesthetized using a mixture of ketamine hydrochloride (100mg/kg) and xylazine (7mg/kg). And then, its head was fixed inside a stereotaxic instrument. The incisor bar was moved to bring the bregma and lambda at the same height. We performed a durotomy (i.e. piercing a tiny hole in the dura) to make a descending path for the microelectrodes. The following four brain areas were selected as regions of interest for acquiring LFPs: left parietal lobe (LP), right parietal lobe (RP), left thalamic (LT) and right thalamic (RT) regions. Stereotaxic coordinates of the target areas (in mm) mentioned in the order (anterior or posterior to bregma, lateral to midline, ventral to dura mater) were as follows: (LP) (−4, +5.5, 0), RP (−4, −5.5, 0), LT (−3, +3, −6.5), and RT (−3, −3, −6.5). Fig 1B shows the brain histology indicating that the deep electrodes reached both the thalami. The implanted microelectrodes (2 skull microelectrodes for parietal lobe and 2 deep microelectrodes for thalamic regions) were tightly fixed to the skull using bone cement. The wound on the scalp was sutured after removing the rat from the stereotaxic frame. After the suture, the rats were placed in a chamber for recovering from the anesthesia and ketoprofen (2.5mg/kg, Uni Biotech, Korea) was injected intramuscularly to reduce pain. After the surgery, antibiotics (Ceftazol 20 mg/kg, Guju Pharma Co, Korea) was also injected intramuscularly for 3 days. All rats had a one-week postoperative recovery period. In addition, we frequently observed the recovery process in this period.

### LFP recording and data acquisition

A week after the implantation of the microelectrodes, we performed LFP recording on rats under isoflurane inhalational anesthesia. The overall protocol of this experiment has been previously described [9]. Briefly, the microelectrodes on the skull were connected to a customized LFP recording device and the acquired LFP was digitized with a Ni-DAQ card (NI PXIe-6368, National Instruments Corp.; 300 Hz sampling frequency). Initially, LFPs from the target areas were measured for 30 minutes in freely moving rats. The acquired LFPs were used as individual baseline references. LFPs were subsequently measured at different levels of isoflurane anesthesia. In order to induce a rapid loss of consciousness (LOC), we anesthetized the rat by delivering isoflurane (3 vol%) to an induction chamber for 3 minutes. We removed the unconscious rat from the induction chamber and placed the mouth of the rat in the nasal cone for delivering the inhalation anesthetic. Following this procedure, we administrated isoflurane at a concentration of 2.5% for 7 minutes. In particular, the first 2 minutes at the 2.5 vol% isoflurane stage were sufficient for reaching a pseudo state-equilibrium between blood plasma and the effective isoflurane concentration. The last 5 minutes at the 2.5 vol% isoflurane stage maintained a stable concentration of the anesthetic. After sustaining 2.5-vol% isoflurane for 5 minutes, the concentration of inhalational anesthetic was decreased to 1 vol% and this concentration was further administrated for 5 minutes. After being exposed to 1 vol% isoflurane for 5 minutes, the rats were allowed to recover from anesthesia for 30 minutes by removing nasal cone from the mouth of the rat. The LFPs in the target areas (i.e. parietal lobe and thalamus) were continuously recorded during the administration of isoflurane with a custom-made software developed in the LabVIEW (LabVIEW 2014, National Instruments Cop.) environment. Before data preprocessing, we chose the LFPs according to the level of anesthetic concentration. In order to analyze the changes of connectivity direction and strength between the regions of interest induced by different anesthesia levels, we selected four different 40 s LFPs, respectively. Since the selected LFP segments were artifact-free, each LEP segment have an information about stable anesthetic concentration. The selected segments were divided into the following four stages: awake (pre-anesthetic period), deep anesthesia (2.5 vol% isoflurane administration), light anesthesia (1 vol% isoflurane administration), recovery (post-anesthetic period) in Fig 1A. Therefore, the total number of segments was 40 (i.e. 10[subjects]×4[stages]).

### Data preprocessing

The selected LFPs from the regions of interest were preprocessed before the application of the connectivity algorithm in order to remove the external noise induced by several external factors. Therefore, we conducted notch filtering to reduce the effect of the 60 Hz line noise generated by commercial electricity. Furthermore, we performed downsampling, i.e. the process of reducing the sampling rate of a signal in order to decrease the effect of added noise originating from the external environment. Since our brain wave range of interest was from 0.5 Hz to 60 Hz, we reduced the sampling rate of LFPs from 300 Hz to 150 Hz. In addition, to enhance the signal-to-noise ratio, we used averaged LFPs from the parietal lobe (P) and thalamus (T). These LFPs were calculated by averaging the signals from left (LP, LT) and right (RP, LT) sides of each area, respectively. Lastly, we divided the 40 s averaged LFPs (P, T) into 20 epochs (20[epochs]×2[seconds]) to apply the connectivity algorithm. The detailed description of epoch length and its effect are described in the next section. Therefore, the total number of epochs is 800 (10[subjects]×4[stages]×20[epochs]).

#### Partial Directed Coherence (PDC)

Partial Directed Coherence (PDC) derived by the multivariate autoregressive model (MVAR) is a popular connectivity method and was defined by Baccala and sameshiam [10]. Unlike Granger causality (GC), which is used to represent the causal relationship between two different signals in the time domain, PDC can describe the direction of information flow between two or more signals in the frequency domain. In other words, PDC extends the concept of directed influence from the time domain to the frequency domain and from two dimensional signals to multidimensional signals. Therefore, PDC is particularly well-suited to analyze brain connectivity using LFPs. Indeed, LFPs consist of multichannel signals and the characteristics of each wave (e.g. gamma, beta, alpha, theta and delta) are highly variable.

In the present study, we assume that the set of Wide Sense Stationary (WSS) signal **x** is estimated from multichannel LFPs and is described as

$$x\left(t\right)={\left[{\text{x}}_{1}\left(t\right),{\text{x}}_{2}\left(t\right),\ldots ,{\text{x}}_{N}\left(t\right)\right]}^{\text{T}}$$ (1)

where *N* and *t* are the number of the LFP channel and the time index, respectively. The superscript T stands for the transpose operation. Let us assume that the MVAR process mentioned below is a proper description of the signal set **x**(*t*)

$$x\left(t\right)=\sum_{l=1}^{p}\text{A}\left(l\right)x\left(t-l\right)+e\left(t\right)$$ (2)

The *p* donated model order of MVAR, residual noise **e**(*t*) = [e_1_(*t*), e_2_(*t*),…, e_*N*_(*t*)]^T^ is the vector of size *N* and **A**(*l*) is the *N*×*N* model coefficients matrix. Since the performance of MVAR depends on the model order *p*, the determination of the appropriate mod’el order *p* can be obtained using an information theory approach [11]. Among the information theory methods available for selecting the proper criteria, we used the Akaike Information Criteria (AIC) for MVAR process [12]. The matrix of model coefficients A(*l*) are calculated by minimizing the residual **e**(*t*). In brief, Eq (2) is transformed to matrix form in the frequency domain and it is described as

$$\;\;\left[\begin{array}{c} \begin{array}{c} {\text{e}}_{1}\left(f\right)\\ {\text{e}}_{2}\left(f\right) \end{array}\\ \begin{array}{c} \vdots \\ {\text{e}}_{N}\left(f\right) \end{array} \end{array}\right]=\left[\begin{array}{cc} \begin{array}{cc} {\text{a}}_{11}\left(f\right) & {\text{a}}_{12}\left(f\right)\\ {\text{a}}_{21}\left(f\right) & {\text{a}}_{22}\left(f\right) \end{array} & \begin{array}{cc} \cdots & {\text{a}}_{1N}\left(f\right)\\ \cdots & {\text{a}}_{2N}\left(f\right) \end{array}\\ \begin{array}{cc} \vdots & \vdots \\ {\text{a}}_{N1}\left(f\right) & {\text{a}}_{N2}\left(f\right) \end{array} & \begin{array}{cc} \ddots & \vdots \\ \cdots & {\text{a}}_{NN}\left(f\right) \end{array} \end{array}\right]\left[\begin{array}{c} \begin{array}{c} {\text{x}}_{1}\left(f\right)\\ {\text{x}}_{2}\left(f\right) \end{array}\\ \begin{array}{c} \vdots \\ {\text{x}}_{N}\left(f\right) \end{array} \end{array}\right]\;e\left(f\right)=\text{A}\left(f\right)x\left(f\right)$$ (3)

where a_*jk*_(*f*), e_*j*_(*f*) and x_*j*_(*f*) are calculated using a Fourier transform. Among them, an element a_*jk*_(*f*) of the model coefficient ***A***(*f*) represents the influence that signal x_*j*_(*f*) exerts on another signal x_*j*_(*f*) in the frequency *f*. The matrix of model coefficient ***A***(*f*) is obtained as

$$\text{A}(f)=\;I\;-\sum_{l=0}^{p}\text{A}(l)e^{-i2\pi fl}$$ (4)

where **I** is a *N*×*N* identity matrix. Eventually, estimated PDC is calculated as

$$\theta_{k\to j}\left(f\right)=\frac{{\text{a}}_{jk}\left(f\right)}{\sqrt{{\sum^{\text{​}}}_{n=1}^{N}{\text{a}}_{nj}\left(f\right){\text{a}}^{*}{}_{nk}\left(f\right)}}$$ (5)

where *θ*_*k*→*j*_(*f*) represents the directional flow of information from *k-*th signal to *j-*th signal in the frequency *f*. Therefore, the result of PDC is a useful index that can be used to analyze the causality between two different signals in the frequency domain. In particular, the PDC process is well-suited to analyze LFPs because these signals have different characteristics depending on the frequency range. However, since the classical PDC based on MVAR is a stationary process, it is not appropriate to apply the classical PDC to a nonstationary signal such as the LFPs because it may produce unexpected results. In order to overcome this issue, the PDC process is applied to LFPs using a narrow window where an optimal window size has been selected [11]. Previous neurophysiological experiments and signal analyses have revealed that the selection of a proper window size for applying MVAR processes can estimate the appropriate stationary stochastic process as well. In the present study, we used a 2 s window size.

#### Statistical analysis

**Fig 2:**
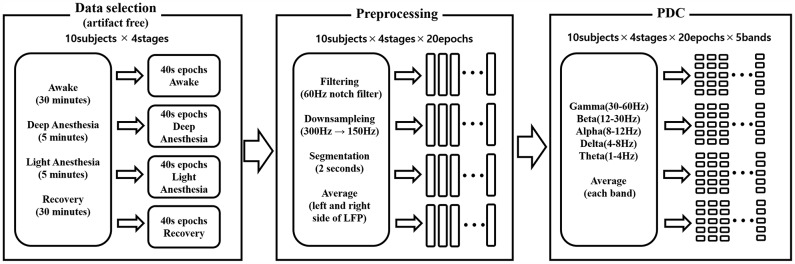
Block diagram for calculating the Partial Directed Coherence (PDC).

The results of PDC were divided in the following frequency bands: delta (1–4 Hz), theta (4–8 Hz), alpha (8–12 Hz), beta (12–30 Hz), and gamma (30–60 Hz) and was averaged for each respective frequency band. Therefore, the total number of PDC feature is 4000 (10 [subjects]×4 [stages]×20 [epoch]×5 [bands]). The overall PDC process structure is represented in Fig 2. The statistical differences in PDC for each frequency band at different anesthetic states were calculated by a Friedman test followed by Dunn’s post-hoc test using a free statistical software R (R 2.13.1) [13]. A P-value less than 0.05 was considered statistically significant.

## Results

**Fig 3:**
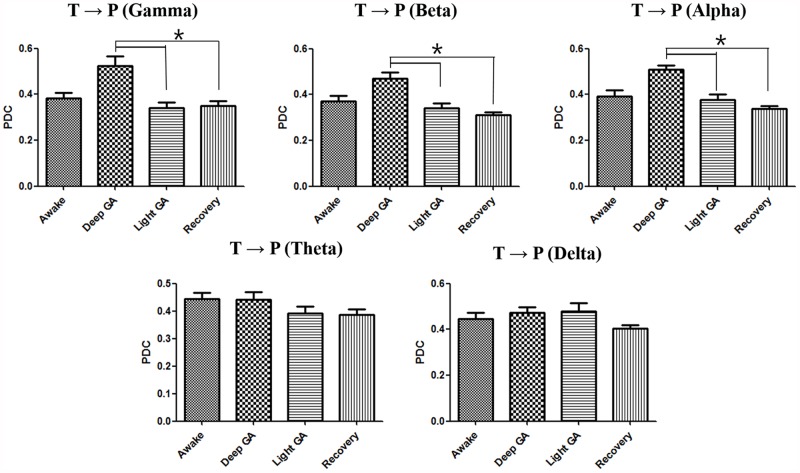
Averaged PDC computed in the thalamus-to-parietal direction at each frequency band according to the anesthesia depth. Error bars indicate standard error. GA: general anesthesia, P: parietal area, T: thalamus.

**Fig 4:**
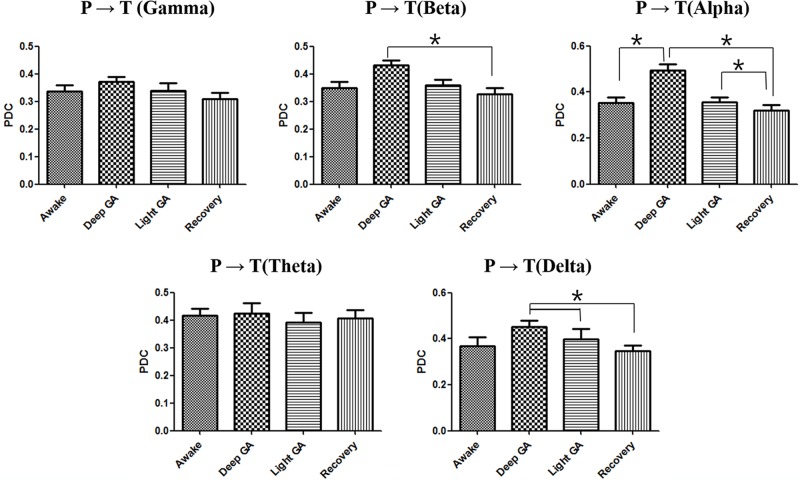
Averaged PDC computed in the parietal-to-thalamus direction at each frequency band according to the anesthesia depth. Error bars indicate standard error. GA: general anesthesia, P: parietal area, T: thalamus.

To confirm the direction and strength of interactions between thalamus and parietal area affected by different levels of anesthesia, PDC was calculated and averaged for all subjects. As shown in Figs 3 and 4, PDC changed at different anesthesia levels for both the thalamus-to-parietal and parietal-to-thalamus directions. The PDC for both directions (thalamus → parietal, parietal → thalamus) showed similar trends as anesthesia depth increased. The PDC in the thalamus-to-parietal direction significantly increased with increasing anesthesia mainly in the high frequency range. PDC increase associated with anesthesia depth changes was most prominent in the alpha frequency band (F = 18.72, P<0.001), suggesting that interactions between the thalamus and parietal areas mainly occur in the alpha frequency range. However, anesthesia-related changes in the parietal-to-thalamus PDC were only significant in in the low frequency range. PDC increase associated with anesthesia depth changes was most prominent in the alpha frequency band (F = 12.77, P = 0.0052), similar to what we observed in the reverse direction. This result suggests that the parietal-to-thalamus interactions mainly occur in the alpha frequency range. The detailed description of change in PDC between parietal and thalamus associated with anesthesia depth changes is present in S1 Table (see supporting information).

**Fig 5:**
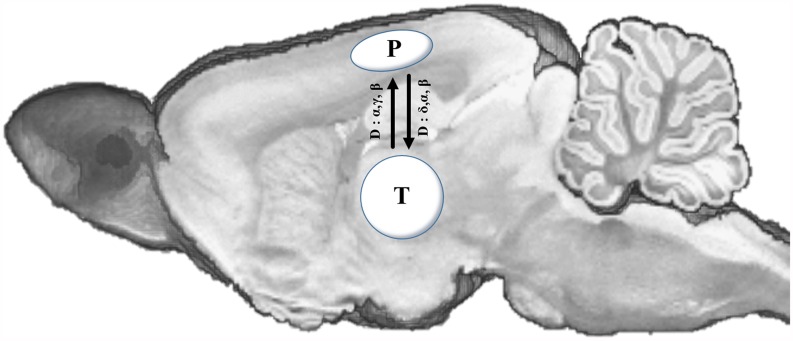
Estimated connectivity of the parietal area and thalamus using PDC. The solid line indicates an increase in connectivity between the two areas. The frequency shown on the line indicates the frequency bands between the two areas that are statistically significant (*P* < 0.05). T: thalamus, P: parietal area.

Taken together, these results suggest that the pattern of connectivity between parietal and thalamic regions is influenced by the depth of anesthesia (Fig 5).

## Discussion

We have shown that PDC preferentially changes in the thalamus-to-cortex direction depending on the anesthesia depth. To the best of our knowledge, this is the first study to investigate large-scale thalamo-parietal interactions across the brain at different levels of anesthesia.

Several lines of evidence have shown that the functional connections between the thalamus and cortical areas are critical in maintaining consciousness. Information flow between the thalamus and the cortex is abruptly changed during the transitional phase of consciousness [2,3]. However, until now, the thalamo-cortical interaction changes induced by anesthesia depth have not been thoroughly investigated. Recently, we have found that different levels of anesthesia have a different impact on the interaction between the thalamus and the frontal cortex, a brain region critical in regulating cognitive function [9]. Our previous findings lead to the hypothesis that anesthesia depth *per se* may affect the functional connectivity between brain areas.

In agreement with our hypothesis and previously reported findings, different levels of anesthesia significantly affect the thalamo-parietal interaction. Considering that the analgesic effect usually increases proportionally to the depth of anesthesia, we predicted that bottom-up sensory processing may be impaired during anesthesia. However, contrary to our prediction, thalamo-parietal synchrony was proportionally increased, rather than being decreased, during anesthesia. Sensory perception can be understood as a hierarchical predictive coding between bottom-up and top down information processing [14–16]. Anesthesia also affects the function of the frontal area, a region that plays a critical role in executive processing [17–18]. If fragmented sensory inputs are not integrated within the framework of sensory information processing, the senses cannot be perceived. Taken together, our results and previous findings lead to the speculation that feedforward connections may be impaired during anesthesia while bottom-up sensory processing appears to be improved.

In this context, we speculate that thalamus-to-parietal bottom-up connectivity may not be reduced because this connectivity is not directed to a high order brain region. However, it is unclear why bottom-up processing is aroused. One conclusion would be that as anesthesia deepens, isoflurane-induced uneven modulation of cortico-thalamic loop might make these robust connections more prominent. Under anesthesia, spontaneous thalamic activity is regulated by feedback connections driven by cortical neuron activity [19]. If feedback connection attenuates the input to the thalamus associated with different anesthetic levels, the inherent and robust connections between thalamus and parietal area may be more prominent.

We have shown that parietal-thalamus interactions are rather increased contrary to frontal thalamus interactions in our previous study. It is not clear why thalamocortical interactions differ from one brain area to another with different anesthetic depths. However, these different results may be explained by considering that sensory stimulation is also an important factor controlling the level of consciousness. Indeed, sensory processing is also affected by modulation of thalamocortical interaction depending on attentional states [20]. Also, it has been known that the detection of complex sound sequences is not significantly impaired in the absence of consciousness [21], suggesting that sensory processing may be more preserved than consciousness processing. Increased thalamo-parietal interaction may be thought as an increase in sensory processing.

However, from the perspective of information integration theory, a synchronized network does not respond efficiently to incoming inputs. A study investigating the mechanisms underlying unconsciousness in generalized epilepsy reported an increase in the synchrony between cortical and subcortical structures [22]. The authors suggested that, on a global neuronal scale, enhanced synchrony paradoxically might block the integration of information relayed from other areas. It has also been reported that connections between thalamic nuclei involved in transferring sensory input from the periphery are relatively maintained while connections between nonspecific nuclei mediating cortical integration are depressed [23]. The parietal lobe receives major sensory inputs from the periphery through the thalamus. Although it is not clear whether the increase in interaction is due to a complementary mechanism that increases arousal or by robust interactions of itself, increased interactions may enhance the paradoxical inherent inter-brain interactions, rendering them less flexible to respond to various types of external stimuli.

The thalamic reticular nucleus is important in regulating the interaction between the thalamus and the cortex [24]. Recent studies have shown that TRN controls sensory processing associated with attention states [20,25]. Isoflurane, a volatile anesthetic, modulates the gamma-aminobutyric acid (GABA) receptors of TRN differently under different anesthetic conditions [26]. The TRN constitue of thin GABAergic neurons [27]. Considering that TRN is important for thalamocortical interaction and regulation of thalamocortical function in relation to sensory processing as well as the function control of thalamic nucleus for sensory processing, the alteration of GABAergic TRN neuron associated with different levels of anesthesia may affect reciprocal thalamo-parietal interaction although a further study warrants to clarify the mechanism of increased thalamocortical interactions from the perspective of neurotransmission.

Interestingly, modulations of thalamo-cortical and cortico-thalamic interactions were statistically significant in the alpha frequency band. Several studies have suggested that brain oscillation in the alpha band may be a core feature of communication between brain areas. The thalamus is known to be involved in generating alpha oscillation [28–30]. Thalamo-cortical circuits are mainly coordinated by an alpha rhythm generated from the thalamus [31,32]. Therefore, it is surprising that reciprocal thalamo-parietal connections increase proportionally with the anesthesia depth in this study. Rather, thalamic activity is augmented during propofol induced ROC unlike depressed cortical activity [33,34]. Taken together, these findings suggest that the robust thalamo-cortical connections in the alpha frequency band are aroused as anesthesia deepens.

Given the large variety of anesthetic agents, it is likely that other types of anesthetics may exert different effects on thalamo-parietal connectivity at different levels of anesthesia. This variability should be taken into account when extending our findings to other types of anesthetics. While our study provides a plausible explanation of what happens to thalamo-parietal connections under different levels of anesthesia, it is unclear whether the increased thalamo-parietal interactions observed under anesthesia is the consequence of a direct action of the anesthetic on the thalamus and cortex or rather this effect is mediated by other brain areas not analyzed in the present study.

In conclusion, we have found that the functional interaction between the thalamus and parietal area is augmented proportionally to the anesthesia levels. The present findings provide a new insight into the processing of sensory inputs from the periphery under different levels of anesthesia. Indeed, the mechanisms underlying this anesthetic-dependent modulation deserve further investigations from the perspective of a global brain network.

## Supporting information

S1 Table Changes in thalamo-parietal connectivity at each frequency band and the time domain depending on anesthetic depth. T: thalamus, P: frontal area, GA: general anesthesia, + indicates statistical significance (P<0.05), ns: statistically insignificant.
(DOCX)
