# Supplementary material for: Differential modulation of thalamo-parietal interactions by varying depths of isoflurane anesthesia
Source: PLoS One. 2017 Apr 6;12(4):e0175191. doi: 10.1371/journal.pone.0175191 (PMC5383263; doi:10.1371/journal.pone.0175191)
Supplement: S1 Table — T: thalamus, P: frontal area, GA: general anesthesia, + indicates statistical significance (P<0.05), ns: statistically insignificant. (DOCX) [file pone.0175191.s001.docx]

**S1 Table.** **Changes in thalamo-parietal connectivity at each frequency band and the time domain depending on anesthetic depth.** T: thalamus, P: frontal area, GA: general anesthesia, + indicates statistical significance (P<0.05), ns: statistically insignificant.

| **Channel Pairs** | **Frequency band** |  | **Awake** | **Deep GA** | **Light GA** | **Recovery** |
| --- | --- | --- | --- | --- | --- | --- |
| **T**$\boldsymbol{\to}$**P** | Delta | Awake | ns | + | ns | ns |
|  |  | Deep GA | + | ns | ns | + |
|  |  | Light GA | ns | ns | ns | ns |
|  |  | Recovery | ns | + | ns | ns |
|  | Theta | Awake | ns | ns | ns | ns |
|  |  | Deep GA | ns | ns | ns | ns |
|  |  | Light GA | ns | ns | ns | ns |
|  |  | Recovery | ns | ns | ns | ns |
|  | Alpha | Awake | ns | + | ns | ns |
|  |  | Deep GA | + | ns | + | + |
|  |  | Light GA | ns | + | ns | ns |
|  |  | Recovery | ns | + | ns | ns |
|  | Beta | Awake | ns | ns | ns | ns |
|  |  | Deep GA | ns | ns | ns | + |
|  |  | Light GA | ns | ns | ns | ns |
|  |  | Recovery | ns | + | ns | ns |
|  | Gamma | Awake | ns | ns | ns | ns |
|  |  | Deep GA | ns | ns | ns | ns |
|  |  | Light GA | ns | ns | ns | ns |
|  |  | Recovery | ns | ns | ns | ns |
|  | **Frequency band** |  | **Awake** | **Deep GA** | **Light GA** | **Recovery** |
| **P**$\boldsymbol{\to}$**T** | Delta | Awake | ns | ns | ns | ns |
|  |  | Deep GA | ns | ns | ns | ns |
|  |  | Light GA | ns | ns | ns | ns |
|  |  | Recovery | ns | ns | ns | ns |
|  | Theta | Awake | ns | ns | ns | ns |
|  |  | Deep GA | ns | ns | ns | ns |
|  |  | Light GA | ns | ns | ns | ns |
|  |  | Recovery | ns | ns | ns | ns |
|  | Alpha | Awake | ns | ns | ns | ns |
|  |  | Deep GA | ns | ns | + | + |
|  |  | Light GA | ns | + | ns | ns |
|  |  | Recovery | ns | + | ns | ns |
|  | Beta | Awake | ns | ns | ns | ns |
|  |  | Deep GA | ns | ns | + | + |
|  |  | Light GA | ns | + | ns | ns |
|  |  | Recovery | ns | + | ns | ns |
|  | Gamma | Awake | ns | ns | ns | ns |
|  |  | Deep GA | ns | ns | + | + |
|  |  | Light GA | ns | + | ns | ns |
|  |  | Recovery | ns | + | ns | ns |
